# Polyphenols as Promising Drugs against Main Breast Cancer Signatures

**DOI:** 10.3390/antiox6040088

**Published:** 2017-11-07

**Authors:** María Losada-Echeberría, María Herranz-López, Vicente Micol, Enrique Barrajón-Catalán

**Affiliations:** 1 Institute of Molecular and Cell Biology (IBMC), Miguel Hernández University (UMH), Avda. Universidad s/n, Elche 03202, Spain; mlosada@umh.es (M.L.-E.); mherranz@umh.es (M.H.-L.); vmicol@umh.es (V.M.); 2 CIBER, Fisiopatología de la Obesidad y la Nutrición, CIBERobn, Instituto de Salud Carlos III (CB12/03/30038), Palma de Mallorca 07122, Spain

**Keywords:** breast cancer, polyphenols, luminal, TNBC, redox balance, apoptosis, autophagy, inflammation, ER, HER2

## Abstract

Breast cancer is one of the most common neoplasms worldwide, and in spite of clinical and pharmacological advances, it is still a clinical problem, causing morbidity and mortality. On the one hand, breast cancer shares with other neoplasms some molecular signatures such as an imbalanced redox state, cell cycle alterations, increased proliferation and an inflammatory status. On the other hand, breast cancer shows differential molecular subtypes that determine its prognosis and treatment. These are characterized mainly by hormone receptors especially estrogen receptors (ERs) and epidermal growth factor receptor 2 (HER2). Tumors with none of these receptors are classified as triple negative breast cancer (TNBC) and are associated with a worse prognosis. The success of treatments partially depends on their specificity and the adequate molecular classification of tumors. New advances in anticancer drug discovery using natural compounds have been made in the last few decades, and polyphenols have emerged as promising molecules. They may act on various molecular targets because of their promiscuous behavior, presenting several physiological effects, some of which confer antitumor activity. This review analyzes the accumulated evidence of the antitumor effects of plant polyphenols on breast cancer, with special attention to their activity on ERs and HER2 targets and also covering different aspects such as redox balance, uncontrolled proliferation and chronic inflammation.

## 1. Introduction

Nowadays, cancer is one of the main causes of mortality worldwide. In 2012, 14 million new cases were diagnosed, and there were 8.2 million cancer-related deaths [1]. Breast cancer is the most common tumor in occidental women; one in eight women will have a breast tumor during their lifetime, and every year, up to 1.4 million new cases are diagnosed worldwide [1]. An annual mortality rate of about 450,000 is estimated, which accounts for 20–30% of all tumors.

Currently, treatments are based mainly on two molecular markers: hormone receptors and epidermal growth factor receptor 2 (HER2). The expression of these molecular markers determines both prognosis and treatment. Although new therapies against breast cancer have been able to reduce mortality, the prognosis, especially in the more advanced stages, remains unpromising, and therefore, further research in this field is needed [2]. The main advances have been obtained for HER2 positive tumors where monoclonal antibodies such as trastuzumab (Herceptin^®^) have improved prognosis in HER2 overexpressing tumors [3]. Unfortunately, even in these cases, resistance is also frequent, leading to nonspecific therapeutic options. In addition, approximately 10–20% of breast tumors are considered triple negative, which implies that no specific therapy is available, and only classic chemotherapy may be applied. 

In this clinical scenario, new efforts have been made to obtain new drugs for breast cancer treatment leading to several promising molecules [4]. However, new molecules are still required. Natural compounds from different origins such as vegetal [5], microbial [6] and marine [7] species are a source of new molecules demonstrating activity against cancer and other diseases. These compounds derive from the secondary metabolism of these organisms and have been selected by nature through evolution. Between natural compounds, polyphenols have emerged as one of the main families of compounds with potential biological activity in many diseases such as cancer [8,9,10,11,12,13], diabetes [14,15], inflammation [16,17,18,19], obesity-related diseases [20], neurodegenerative disorders [21,22,23], bacterial [24,25,26,27] and viral infections [28,29] or cardiovascular diseases [30]. In addition, they possess a relevant antioxidant activity [24,31,32,33,34,35], which is the basis of part of their biological activity. 

**Figure 1:**
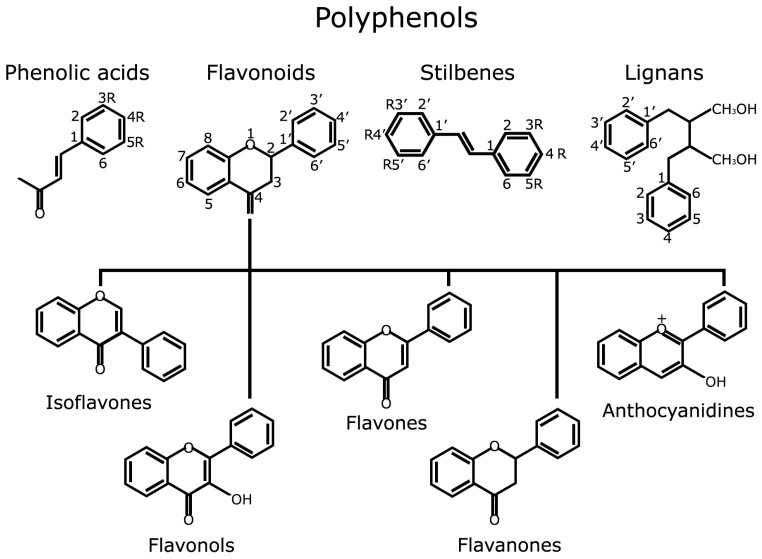
Polyphenols structure and classification.

Polyphenols are widely distributed in fruits, vegetables, tea, essential oils and cereals; their molecular structure is characterized by the presence of one or more phenolic rings substituted with at least one hydroxyl group. Different classes and subclasses of polyphenols generate a large structural variability that is characterized by the number of phenolic rings they possess and the moieties that substitute their aromatic rings (see http://phenol-explorer.eu/compounds/classification for an updated classification). The main groups of polyphenols are: phenolic acids, flavonoids, stilbenes and lignans (Figure 1).

As mentioned above, some polyphenols have demonstrated anticancer activity, showing biological activity against most of the main cancer molecular targets such as kinases, pre- and anti-apoptotic proteins, enzymes that regulate energy metabolism and regulatory proteins linked to proliferation and signaling pathways [36]. Their broad activity may be attributed to several mechanisms, including the interaction and modulation of a wide range of proteins, enzymes and membrane receptors, regulation of gene expression, apoptosis induction, vasodilation and modulation of cell pathways [37,38,39,40,41,42]. In addition, polyphenols have preventive effects against tumor initiation through numerous mechanisms, such as the avoidance of genotoxic molecule formation, the blockade of mutagenic transforming enzyme activity [43], the regulation of Phase I and II enzymes, such as cytochrome P450s (CYP) [44] and S-transferase (GST) [45], as well as preventing DNA damage [46,47]. 

For all these reasons, new treatments based on polyphenolic compounds are being studied as an alternative and/or adjuvant therapies in these pathologies using different models [21]. Potential benefits of their dietary intake on human health and, more specifically, on cancer risk (including breast cancer) have been also reviewed [48,49]. Specifically, for breast cancer, interesting results have been obtained with a mixture of tea extract and quercetin [50], with *Pinus radiata* [51], Indian lotus [52], *Hypogymnia physodes* lichen [53], *Morinda citrifolia* [54] or with olive leaf extracts [55,56,57,58], among others.

This review describes the different breast cancer types, molecular biomarkers and their main treatments. A compilation of the main molecular breast cancer targets and the use of polyphenols to address them is reviewed, covering different aspects such as redox balance, uncontrolled proliferation and chronic inflammation, with particular interest in ER (estrogen receptor) and HER2 and the use of polyphenols to modulate their pathways.

## 2. Breast Cancer Biomarkers Determine Both Prognosis and Treatment

The identification of molecular biomarkers plays a significant role in the diagnosis and prognosis of breast cancer. They represent therapeutic targets, and their expression is used to classify cancers according to the different molecular subtypes (Table 1). The major biomarkers of breast cancer include the hormonal estrogen and progesterone receptors (ER and PR) and HER2/ERBB2 and, with less relevance, Ki-67 protein [59]. These markers have been extensively studied, and their expression correlates with differences in tumor behavior and patient response to treatments [60,61]:

- Hormone receptors, ERs and PR, are the main factors responsible for hormone response. Breast cancer is a hormone-dependent tissue, and this response is controlled by these receptors [62,63]. ER and PR expression confer a better prognosis and are the basis of hormonal therapy.
- HER2 is a membrane receptor involved in cell proliferation signal transduction. It is present in normal cells and in most tumors, but in 5–15% of breast tumors is overexpressed, increasing tumor aggressiveness [59]. These tumors are very often sensitive to treatment with anti-HER2 treatments, such as humanized monoclonal antibodies or specific inhibitors [64].
- Ki-67: is a protein marker that can be only detected in proliferating cells and currently is used to rate tumor proliferation, particularly lymphomas, breast, endocrine and brain cancers [65]. Indeed, Ki-67 contributes greatly to the Oncotype score [66]. Tumors with high proliferation rates (>15%) have a poor prognosis [65].

Using these molecular markers, breast cancer can be divided into four major molecular subtypes: Luminal A, Luminal B, HER2 type and triple negative breast cancer (TNBC) [67,68,69]. This division determines treatment as shown in Table 1. Overexpression of HER2 is related to the lack of expression of hormone receptors in most cases. The same situation occurs with Ki67, which is usually elevated in cells that do not express these receptors. Between Ki67 and HER2, no relationship has been found.

Luminal A tumors are hormone dependent, with hormone receptors positive expression (ER/PR-positive). They are HER2 negative and present one or two tumor grades. They represent 30–70% of breast cancers [70,71,72] and have the best prognosis, with high survival and low recurrence rates [73,74,75].

Luminal B tumors tend to be ER/PR-positive. They can be HER2-negative or positive; in this last case, some authors consider it as a new sub-type called Luminal C [76], but this classification is not widespread. They are also characterized by a higher tumor grade, a larger tumor size and a positive lymph node dissemination. Patients with Luminal B tumors are usually diagnosed at more advanced ages than in cases with Luminal A [75,77]. Compared to Luminal A tumors, they also tend to have factors that lead to a poorer prognosis, mainly an increase in Ki67 protein of 15–20% [78,79]. The prevalence of Luminal B tumors is approximately 10–20% [70,71,72] and still shows high survival rates, although not as high as those of Luminal A tumors [75]. Treatments for both Luminal A and B are based on hormone therapy regimens [80], which is based on the use of SERMs (selective estrogen receptor modulators) such as tamoxifen or fulvestrant and aromatase inhibitors like anastrozole, exemestane and letrozole [81].

HER2-type tumors are characterized by being ER/PR-negative, overexpressing HER2, having lymph node positive implication and present poorer tumor grade [74,75]. Patients with HER2 tumors are usually diagnosed at an earlier age than Luminal A and Luminal B [75]. Approximately 5–15% of breast cancers are HER2-positive [71,72] and can be treated with specific anti-HER2 drugs. This group includes monoclonal antibodies like trastuzumab and pertuzumab and specific HER2 inhibitors like lapatinib [64]. Before these drugs were available, HER2-type tumors had a rather poor prognosis [71,82].

TNBC encompasses all tumors that are negative for ER, PR and HER2. It is considered the most metastatic type of breast cancer and has highly invasive properties, is larger, has a poorer prognosis with a high probability of relapse, no response to hormonal therapy and has nodal involvement. Around 10–20% of tumors correspond to triple negative tumors [70,71,72]. There are several approaches to counteract TNBC, but all based on classical chemotherapy using anthracyclines, taxanes, poly(ADP-ribose) polymerase protein inhibitors and platinum-containing chemotherapeutic agents [83].

## 3. Breast Cancer Signatures and Polyphenols

### 3.1. Redox Balance

Oxidative stress is caused by an imbalance between the production of reactive oxygen species (ROS) and the efficacy of the endogenous antioxidant system. Tissues are continuously exposed to free radicals derived from metabolism or due to external factors such as pollution or radiation [84,85,86]. In fact, ROS participate in physiological functions such as metabolism signaling and defense against infections [87,88]. However, uncontrolled ROS production or accumulation can induce lipid peroxidation, protein modifications and DNA damage. These events lead to membrane alterations, protein dysfunctions and genetic alterations, all of which are linked to carcinogenesis and tumor progression. 

Balance between oxidant species and antioxidants (redox balance) is essential to maintain a healthy cell status. Breast cancer is characterized by a systemic prooxidant status [89], and an increased ROS presence is determinant for some relevant events such as tumor progression mediated by stromal cells [90]. However, ROS can play a dual role [91], not only in breast [92], but in all cancers. Cells have several mechanisms to transform and eliminate ROS and avoid their harmful effects, such as superoxide dismutase (SOD), catalase (CAT) or glutathione peroxidase (GTX) enzymes [93,94,95]. The synergistic action between these enzymes, vitamins and exogenous antioxidants such as polyphenols allows neutralizing free radicals and modulating cellular signaling [96].

Initially, increased ROS production leads to a pro-oncogenic situation as it provokes two crucial effects: mitochondrial dysfunction that conduces to protein oxidation, lipid peroxidation and DNA damage. On the other hand, once tumor cells have developed, an increase in ROS presence can lead to tumor cell death. This fact has been linked to some anticancer drugs such as doxorubicin and paclitaxel.

Polyphenols can participate in these two situations. First, it is generally admitted that polyphenols are antioxidants and therefore counteract ROS production and inhibit oxidative DNA damage and mitochondrial dysfunction by acting as chemo-preventive agents [97,98]. For this reason, the preventive character of polyphenols acquired through diet in diseases such as cancer [99,100], diabetes [101,102] or atherosclerosis [103,104] has been studied. 

However, there is increasing evidence suggesting that under certain conditions, polyphenols can act as prooxidants, leading to tumor cell death [105,106]. For example, it has been shown that in systems containing active redox metals such as copper, some polyphenols show prooxidant activity, catalyzing their redox cycle and leading to ROS formation [107,108]. Since copper levels in cancer cells compared to healthy cells are increased [109], this prooxidant mechanism would present preferential cytotoxicity against cancer cells, leaving the normal cells undamaged. This effect has been demonstrated using polyphenols like luteolin, apigenin, epigallocatechin-3-gallate and resveratrol [110].

### 3.2. Uncontrolled Proliferation

One of the main characteristics of tumor cells, no matter their origin, is their ability to grow and proliferate in an uncontrolled way. Cellular proliferation is mainly linked to cell cycle progression. This cycle shows different checkpoints in which the cell examines the internal and external signals and decides whether to proceed with cell division or not. This uncontrolled cell division is caused by a malfunction of these checkpoints in the cell cycle [111,112]. The most important regulators of the cell cycle are proteins called cyclins, enzymes called cyclin-dependent kinases (CDKs) and anaphase-promoting enzymatic complex (APC/C) [113,114]. In addition, the active CDK complexes are regulated by binding to CDK inhibitors (p21 and p27) and by other kinases and phosphatases, which control the cell cycle by balancing CDK activity. Variations in the concentration of these inhibitors can alter the normal sequence of the cell cycle as occurs in some tumors or in aged cells.

Polyphenols can act by modulating cyclins, Cdks or APC/C causing cell cycle arrest [115,116]. This cytostatic activity has been also studied in breast cancer, where some polyphenolic compounds have demonstrated their cytostatic activity. For example, ginnalins A–C induce cell cycle arrest in the S and G2/M phases in colon cancer HCT-116 cells and breast cancer MCF-7 cells by decreasing cyclin A and D1 levels [117]. Green tea polyphenols induce cell cycle arrest at G1/G0 phase in breast cancer MCF-7 cells [118]. Polyphenolic extracts from hawthorn fruit have shown a cytostatic effect on MCF-7 breast cancer cells by blocking the cycle in S phase [119]. Ellagic acid induces cell cycle arrest at G0–G1 in human breast cancer MCF-7 cells mediated by a cyclin A2 and cyclin E2 downregulation and an upregulation of the CDK-inhibitors *p*21^Cip1^, *p*15 and *p*19 [120].

In addition to cytostatic activity, however, uncontrolled proliferation can be also treated through cytotoxic mechanisms like apoptosis, autophagy, necrosis or necroptosis. Polyphenols can participate in all these mechanisms as described below.

#### 3.2.1. Apoptosis

Apoptosis is the main mechanism of cell-programmed death. It is characterized by a series of molecular processes that result in cell membrane blebbing, nuclear and chromosomal DNA fragmentation, chromatin condensation, fragmentation and the translocation of phosphatidylserine to the outer face of the plasma membrane, which means that they are eliminated by macrophages [121,122,123].

There are two different apoptotic pathways, mainly differentiated by their starting stimuli. These two pathways may overlap and share some molecular targets as caspases [121]. On the one hand, the extrinsic pathway is mediated by ligands that bind to receptors on the cell surface. Alternatively, the intrinsic pathway is mediated by cellular stress or by DNA injury. There are several molecular mechanisms that tumor cells use to suppress apoptosis. For example, tumor cells may acquire resistance to apoptosis by downregulation of anti-apoptotic Bcl-2 expression or mutation of the pro-apoptotic BAX protein. Expression of both is regulated by the tumor suppressor gene p53 [124], which is mutated in a large number of cancer types [125]. 

As occurred in previous sections, polyphenols can act over different stages of apoptosis. For example, anti-apoptotic Bcl-2 expression is decreased in human breast cancer cell lines, MCF-7 and T47D with a silibinin treatment [126]. Besides, silibinin upregulated phosphatidylinositol-3,4,5-trisphosphate-3-phosphatase (PTEN) and caused a slight increase in p21, thus promoting apoptosis [127,128]. The treatment with Annurca apple polyphenolic extract increases the levels of p53, p21 and the pro-apoptotic ratio of Bax/Bcl-2 in parallel with caspase-9, -6 and -7, in MCF-7 breast cancer cells [129]. Fruit peel polyphenolic extract from different sources (red grape, blackberry, black cherry, black currant, elderberry, blackthorn and plum) induced caspase-dependent cell death associated with an increase in oxidative stress, causing the release of pro- and anti-apoptotic mitochondrial proteins from the Bcl-2 family in breast cancer MCF-7 cells [130]. Oleuropein induced apoptosis due to upregulation of both p53 and Bax gene expression levels and downregulation of Bcl2 in human breast cancer MCF-7 cells [131] and in the HepG2 human hepatoma cell line [132].

Tea polyphenols such as epigallocatechin gallate (EGCG) downregulate telomerase activity in breast cancer cells thereby increasing cellular apoptosis and inhibiting cellular proliferation of MCF-7 and MDA-MB-231 breast cancer cells [133]. They can also inhibit cell growth and induce apoptosis through downregulation of survivin expression, a member of the inhibitor apoptosis protein family (IAP) that inhibits caspases and blocks cell death [134]; this effect has been observed in MCF-7, SK-BR-3 cells and MDA-MB-231 breast cancer cell models [135,136]. 

Artichoke polyphenols modify Bcl-2 and BAX expression in human breast cancer cell line MDA-MB-231, leading to a pro-apoptotic situation [137], which was accompanied with the upregulation of p21 [138].

Finally, other widely-distributed polyphenols such as resveratrol [139], quercetin [140] and catechin [141], promote apoptosis by decreasing IAP1 and survivin expression and increasing FAS ligand and its receptor [142] expression.

#### 3.2.2. Autophagy

Initially, autophagy or cellular autodigestion is a route involved in the degradation of proteins and organelles that may be important in the pathogenesis of some diseases. Dysfunctions in the autophagy process are associated with cancer [143,144,145], neurodegeneration [146,147], aging [147,148] and infections [149,150]. The major proteins involved in the regulation of autophagy are the mammalian target of the rapamycin (mTOR), phosphatidylinositol 3 kinase (PI3K), AKT kinase (AKT), Beclin-1 and p53 [151]. Activation of the PI3K/AKT pathway implies mTOR activation, leading to autophagy inhibition [152]; on the contrary, mTOR inhibition is related to autophagy activation. In addition to mTOR, there is a family of proteins called ATG (autophagy-related protein), such as LC3, ATG5 or ATG12, that are also involved in the regulation of this mechanism.

Some polyphenols can induce tumor cell death through autophagy activation; for example, *Solanum nigrum* L. extract decreased p-AKT levels causing mTOR inactivation and triggering autophagy in AU565 human breast cancer cells [153], as well as blueberry polyphenols in MDA-MB231 cells [154] and grape skin extracts in a murine model of breast cancer [155]. Mango polyphenols cause downregulation of mTOR in human breast ductal carcinoma in situ xenograft models [156].

Resveratrol increases levels of LC3 and its lipidic form, LC3-II, which induces autophagy [157] in human breast cancer MCF-7 cells [158], as well as polyphenol-enriched extract of *Pimenta dioica* berries does in human breast cancer MCF-7, MDA-MB231, SkBr3, BT474 and T47D cells [159] and carnosol in MDA-M231 cells [160]. In another study, it was observed that LC3-II, Beclin 1 and Atg 7 were significantly upregulated by resveratrol [161], inducing autophagy. 

### 3.3. Chronic Inflammation and Pro-Inflammatory Factors

Chronic inflammation has been linked to the development of some tumors [162,163,164], and it is known that diseases such as pancreatitis [165], hepatic steatosis or Crohn’s disease [166], which present chronic inflammation, significantly increase the risk of cancer. This direct relationship with cancer development is also observed in infectious diseases that produce inflammation, such as hepatitis [167,168] or stomach infection by *Helicobacter pylori* [169,170,171]. 

Cell signaling by inflammatory cytokines had been shown to promote the development of cancer [172,173]. However, it was not until 2008, when the direct link between inflammation and cancer was first established, when it was observed that chronic inflammation causing DNA damage led to cancer development [174].

The abilities to inhibit or block the activity of NF-kB [175,176], cyclooxygenase (COX-2) [176,177,178] and lipoxygenase (LOX) [178,179] are the main causes of the anti-inflammatory capacity observed for polyphenols; thus, the role of phytochemicals in these pathways and in cancer-related inflammation has been extensively studied [180]. In this regard, individual polyphenols such as curcumin, EGCG and resveratrol have been reported to show anti-inflammatory effects [180], most of them by reducing NF-κB activation or expression. In addition to individual compounds, cocoa polyphenols decreased the nuclear levels of NF-κB and the expression of pro-inflammatory enzymes such as COX-2 and inducible NO synthase. Additionally, cocoa polyphenols effectively downregulated the levels of inflammatory markers induced by tumor necrosis factor-alpha (TNF-α) by inhibiting NF-κB translocation and JNK phosphorylation [181]. 

Pomegranate polyphenols also demonstrated anti-inflammatory activity as they were able to decrease NF-κB [182] and Nrf-2 [183] pathways in breast cancer cells. Cranberry extract also showed the ability to modulate PI3K/AKT, MAPK/ERK and STAT3 pathways, central nodes in the inflammatory signaling of cancer stem cells [184].

### 3.4. ER and Estrogen Synthesis

Estrogens exert their biological action by binding to ER-α and ER- β, both of which are members of the nuclear receptor superfamily of transcription factors. Although both ERs can develop estrogen-related responses, ER-α is the one that plays an important role in breast cancer (in this review, the term “ER” is used for “ER-α”). 

**Figure 2:**
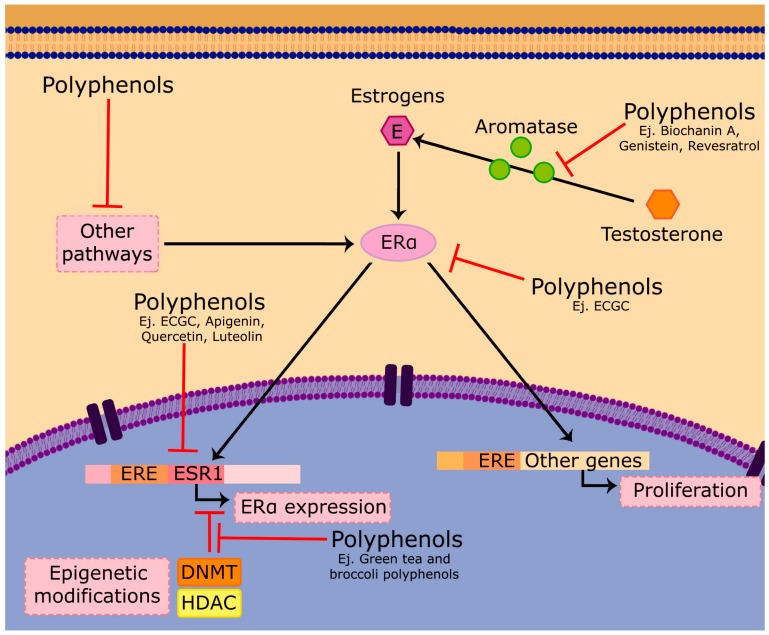
Estrogenic response can be modulated by polyphenol activity. ER activation leads to cell proliferation by activating estrogen response element (ERE)-controlled genes, including their own ER gene (ESR1). Some polyphenols are able both to inhibit ER activity (antiestrogenic action) or ER expression and consequently reduce cell proliferation. The ER activity can also be regulated by some transactivation pathways including the Ras/Raf, PI3K/Akt, AMPK or PKC pathways; all of them can be also modified by polyphenols. ESR1 gene expression can be also regulated by epigenetic modifications through chromatin modifying enzymes such as DNMT (DNA methyltransferases) and HDAC (histone deacetylases). Finally, estrogen synthesis from androgens by aromatase can be also blocked by some polyphenols.

The effects of polyphenols on ER and estrogen pathway is mainly based on three actions: anti-estrogenic mechanisms [185,186,187], changes in ER expression and aromatase modulation [188,189,190] (Figure 2). Anti-estrogenic mechanisms are based on the structural similarities between some groups of flavonoids, such as isoflavones and lignans, and estrogens. In fact, these compounds are considered as phytoestrogens [191,192,193,194,195,196,197] due to their natural origin and their ability to interact with ERs. Polyphenols may exert different effects according to the dose administered. Low dose treatments have an activating effect on the estrogen receptor, which triggers the proliferative response favoring tumor development. Conversely, high concentrations promote processes such as apoptosis or cell cycle blockage resulting in antitumor effects as mentioned above. In addition, ER expression is also regulated by some polyphenols, for example, EGCG, which downregulates ER-α protein, mRNA and gene promoter activity in MCF-7 [198] and ER and PR in T-47D [199] cell lines. There are so many additional examples of flavonoids, like apigenin, luteolin myricetin, anthocyanidins or quercetin, and other compounds, such as stilbenes, ellagitannins, sulforaphanes, curcuminoids and tocopherols, among others, that are extensively reviewed [197,200].

Aromatase enzyme, a member of the CYP superfamily of enzymes, is encoded by the CYP19 gene and supposed to be another target of breast cancer. Its function is to aromatize androgens, producing estrogens. One strategy to counteract the proliferative effect of estrogen in breast cancer is to use aromatase inhibitors. Some polyphenols have shown aromatase inhibiting properties like biochanin A, an isoflavone extracted from red clover, and genistein, which have been reported to inhibit the activity of the aromatase enzyme in SK-BR3 [201]. Resveratrol also inhibits aromatase by significantly reducing the CYP19-encoding mRNA abundance in SK-BR-3 cells [202]. Isoliquiritigenin inhibited aromatase mRNA expression and suppressed the activity of CYP19 promoters I.3 and II in MCF-7 cells [203].

Besides the above-mentioned mechanisms, some polyphenols are able to transform ER-negative phenotypes into ER-positive, thereby improving prognosis and allowing treatment with SERMs such as tamoxifen. This transformation may be due to epigenetic modifications mediated by chromatin modifying enzymes such as DNMT (DNA methyltransferases) and HDAC (histone deacetylases) [204,205,206]. Methylation of the ESR1 gene encoding for ER is associated with the ER-negative phenotype and therefore supports the hypothesis that treatment with DNMT inhibitors and HDAC activators can promote ER expression by reversing the ER-negative phenotype to ER-positive [207]. Green tea polyphenols have been shown to modify chromatin by inhibiting DNMTs and eliciting ER expression in the TNBC MDA-MB231 cell line [208]. It has also been observed that with a combined treatment of green tea extract and broccoli shoots, the epigenetic reactivation of ER is triggered, which in turn increases the sensitivity to tamoxifen in ER-negative breast cancer MDA-MB-231 and MDA-MB-157 cells.

### 3.5. HER2/ErRB Overexpression

**Figure 3:**
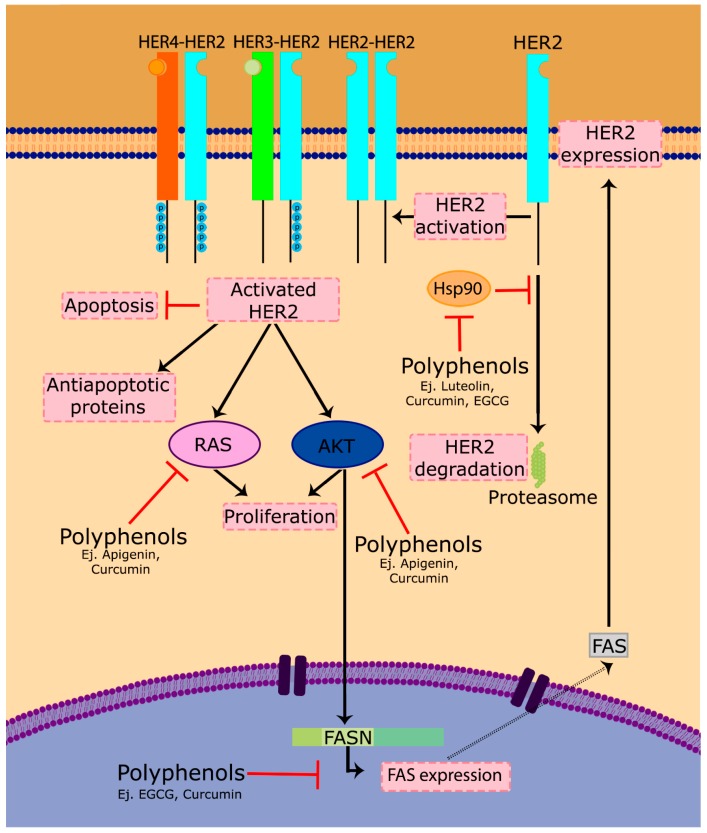
Polyphenols modulate the HER2 pathway. The HER2 membrane receptor, when activated, dimerizes with other HER2 (homodimerization) or other HER family receptor (heterodimerization) and transduces proliferation signals to downstream pathways such as the Ras/Raf or PI3K/Akt routes. This activation also inhibits apoptotic signals and promote antiapoptotic mechanisms. In addition, HER2 expression can be modulated by FASN, and some polyphenols are able to modify this action by actuating on FASN activity and/or expression. Finally, Hsp90 chaperone is required for HER2 to be functional, and some polyphenols modify this interaction leading to HER2 degradation by proteasome.

HER2, also termed ERBB2 or EGFR2, is overexpressed in more than 30% of breast cancers and has been shown to play an important role in the progression of certain aggressive breast cancers. HER2 is a trans-membrane receptor tyrosine kinase that activates multiple proliferative signaling pathways, including PI3K/Akt and Ras/MAPK. Upon activation, HER2 can both homo- or hetero-dimerize with other HER family receptors, leading to signal transduction and cell proliferation (Figure 3). This is the main reason for which HER2 overexpression conduces to an abnormal proliferation and cancer spread. In addition to this main mechanism, however, it has also been shown that several apoptotic mechanisms are deregulated in cells overexpressing HER2 [209,210], and some anti-apoptotic proteins including Bcl-2, Bcl-xL and Mcl-1 are overexpressed [211]. These two additional mechanisms contribute to tumor cell survival and the spread of the cancer (Figure 3).

Nowadays, the most commonly-used therapy against HER2-positive tumors is the monoclonal antibody trastuzumab [212]. This treatment has positively revolutionized the prognosis of this kind of tumor; however, the main problem presented by these patients is the development of resistance to trastuzumab. Polyphenols can sensitize HER2-positive cells through different mechanisms: by decreasing its activation or downloading its expression. For example, some secoiridoids such as oleuropein promote a downregulation of HER2 by blocking ATP binding to the tyrosine kinase domain of the protein [213,214]. Silybin and silybin-phosphatidylcholine exhibit dose-dependent cell growth inhibitory effects and downregulation of HER2 in SkBr3 cells [215]. Apigenin induces apoptosis by depletion of HER2 protein and, in turn, suppression of signaling of the HER2/HER3-PI3K/Akt pathway [216]. HER2, p-Akt, p-MAPK and NF-κB oncoprotein levels decreased in a dose- and time-dependent way in BT-474, MCF- 7, MDA-MB-231 and SkBr3-hr (a herceptin-resistant strain from SkBr3 breast cancer cells) when treated with curcumin [217].

On the other hand, HER2 needs to interact with Hsp90 and its chaperone to acquire its function [218,219], so another possible therapeutic approach is the use of inhibitors of the HER2-Hsp90 binding, causing the dissociation of HER2 from its chaperone and, consequently, leading to HER2 degradation by the proteasome [220,221]. It has been observed that some polyphenols such as luteolin [222], geraniin [223], curcumin [224] or EGCG [225] are inhibitors of Hsp90 and therefore affect the HER2-Hsp90 binding.

Fatty acid synthase (FASN) has been linked with several types of cancer [226]. Overexpression of HER2 has been shown to increase FASN translation, which alters the activity of the mTOR and the PI3K/AKT signaling pathway in breast cancer cells [227]. FASN inhibition induces apoptosis and creates cytotoxicity [228,229,230], but also causes a marked decrease in the active forms of the HER2 protein, indicating that the HER2 oncogene presents positive feedback with FASN to ensure de novo overactive biogenesis of fatty acids. Consequently, FASN inhibition correlates with an inhibition of HER2 (Figure 3). Some polyphenols, such as curcumin [231], some olive oil lignans [232] and EGCG [233] have shown anticancer activity on HER-positive models through this FASN-linked mechanism.

## 4. Conclusions

Breast cancer is a heterogenic oncological disease in which, in addition to common cancer signatures, estrogenic response and HER2 expression constitute two clinical and molecular targets that must be taken into account. 

As stated in this review, polyphenols are natural compounds that have demonstrated antitumor activity reaching different molecular targets. Some of them, especially EGCG, curcumin and flavonoids, such as luteolin, apigenin or quercetin, seem to be the most promising ones according to the current studies. Their promiscuous activity against different molecular targets is quite relevant and suggests that multitargeting therapy can be a future strategy for breast cancer treatment. However, additional aspects such as polyphenols’ bioavailability and toxicity must be addressed. Polyphenols’ bioavailability has been thoroughly studied for many polyphenols, especially those with the most relevant biological activity [234]. In general, polyphenols’ bioavailability is low, and most of them are classified as Class IV by the Biopharmaceutical Classification System (BCS) (low permeability, low solubility) [235]. However, numerous studies have explored strategies to increase polyphenols’ bioavailability, for example using different encapsulation techniques [236,237,238,239] or using pharmaceutical dispersions [240]. Intravenous administration represents an option for many anticancer drugs with poor bioavailability, so this could be a solution too for those polyphenols showing low bioavailability. 

Toxicity is, as mentioned above, another relevant aspect to be studied. As occurred with their bioavailability, polyphenols’ toxicity has been deeply studied [241,242,243], and in most cases, only very high doses present relevant toxic consequences. Moreover, new approaches to predict toxicity using bioinformatic tools such as admetSAR and DataWarrior [244,245] are also available, and new alternative methods both reducing and/or replacing animal use have been introduced in preclinical research (see http://www.oecd.org/chemicalsafety/testing/animal-welfare.htm). However, toxicity should not be a major problem since polyphenols are less toxic than most anticancer drugs.

All the accumulated evidence on the in vitro anticancer activity of polyphenols, as well as bioavailability and toxicity studies may be used to develop new anticancer drugs. Nevertheless, much preclinical research needs to be conducted before administration to oncologic patients with the required guaranties. In vivo studies and, especially, human clinical trials must be developed before real clinical use. However, most of these polyphenols can be obtained from the diet, allowing preventive or nutritional strategies that, although they must be challenged with clinical trials before being accepted, can be implemented in the short term as dietary recommendations. Some studies have been already conducted [48,49], but there is still a need for more evidence.

Future research must be focused on providing new and strong evidence about polyphenols’ activity on every breast cancer signature. As mentioned above, preclinical and clinical evidence is scarce. New studies using omic sciences and in silico approaches could provide some of the pending results as occurred in other disciplines. Genomics, transcriptomics, proteomics, metabolomics and fluxomics strategies are continuously increasing our knowledge about polyphenols’ mechanism of action [246,247]. In silico techniques, such as molecular docking, are being used to identify new ligands for specific cancer molecular targets [248,249]. Epigenetic studies are also providing relevant information [250,251] on polyphenols’ effects, but a global approach using all the updated knowledge is undoubtedly the main pending task. 

## Figures and Tables

**Table 1:** Breast cancer molecular subtypes and their main treatments. Representative cell lines for each subtype are also shown.

Subtype	ER/PR	HER2	Ki67	Treatment	Cell lines
Luminal A	+/+	−	<15%	Antihormonal	MCF7, T47D
Luminal B	+/+	−/+	>15%	Antihormonal	BT474
HER2-type	−/−	+		Anti-HER2	SkBr3, AU565
TNBC	−/−	−/−	>15%	Chemotherapy	MDA-MB231

## Abbreviations

HER2/ERB2	human epidermal growth factor receptor 2
CYP	cytochrome P450s
GST	s-transferase
TNBC	triple negative breast cancer
ER	estrogen receptor
PR	progesterone receptor
ROS	reactive oxygen species
SOD	superoxide dismutase
CAT	catalase
GTX	glutathione peroxidase
CDKs	cyclin-dependent kinases
APC/C	anaphase-promoting complex
PTEN	phosphatidylinositolo-3,4,5-trisphosphate-3-phosphatase
EGCG	epigallocatechin gallate
IAP	inhibitor apoptosis protein
mTOR	mammalian target of the rapamycin
PI3K	phosphatidylinositol 3 kinase
AKT	AKT kinase
COX-2	cyclooxygenase
LOX	lipoxygenase
TNF-α	Tumoral Necrosis Factor-alpha
DNMTs	DNA methyltransferases
HDACs	histone deacetylases
FASN	fatty acid synthase
